# Buffet Cattle Cars

**Published:** 1897-06

**Authors:** W. P. Bass


### Buffet Cattle Cars.

While improvements are being made in passenger equipment, it must not be forgotten that the live stock car companies are not going to sleep; at least there is one up in Chicago representing the Street company that is keeping up with the procession. In a late issue of the *Fort Worth Gazette*, his communication appears in the following original language:

DEAR SIR:—I have billed you one of our new cars for inspection of shippers, carded for you at Fort Worth. This car has all the latest improvements, and I think will be exactly what shippers want for handling their cattle. We have not included hot and cold gas, because there would be danger of suffocating the cattle if a green steer should leave the gas turned on recklessly over night. We have no barber shop, because we think the prairie winds will blow the whiskers off the steers, and they will reach market pretty clean anyhow. In reference to the buffet service, if the shippers choose, where they have weak cattle, they can fill the racks with asparagus and the troughs with mock turtle soup, and this will remove the freckles from the faces of the steers, curl their hair, and bring the rosy flush of health to their cheeks.

Yours truly,

W. P. BASS, A. G. M.

